# Darboux transformation and quasideterminant solutions of a noncommutative semi-discrete coupled dispersionless integrable system

**DOI:** 10.1038/s41598-026-56247-5

**Published:** 2026-06-04

**Authors:** H. W. A. Riaz, Yakup Yildirim, Tariq Aljaaidi

**Affiliations:** 1 School of Civil and Hydraulic Engineering, Ningxia University, Yinchuan, PR China; 2 Center for Theoretical Physics, Khazar University, 41 Mehseti Str., AZ1096 Baku, Azerbaijan; 3 Department of Computer Engineering, Biruni University, 34010 Istanbul, Turkey; 4 Mathematics Research Center, Near East University, 99138 Nicosia, Cyprus; 5 Mathematics Unit, Department of Computer Science, College of Computer and Information Technology, Al-Razi University, Sana’a, Yemen

**Keywords:** Noncommutative integrable systems, Quasideterminants, Darboux transformation, Semi-discrete coupled dispersionless system, Solitons, Mathematics and computing, Physics

## Abstract

We present a noncommutative generalization of the semi-discrete coupled dispersionless integrable system. The motivation stems from the role of noncommutative geometry in string theory and D-brane dynamics, and the need to understand soliton dynamics in matrix-valued field theories beyond the standard abelian setting. A matrix Lax pair for the noncommutative semi-discrete coupled dispersionless system is proposed, and the corresponding equations of motion are obtained as the compatibility conditions of this Lax pair. A Darboux transformation is constructed both for the Lax pair and for the nonlinear field equations, and its iteration yields multisoliton solutions written in a compact quasideterminant form. We then investigate noncommutative semi-discrete solutions of the matrix fields and discuss their qualitative behaviour on the space–time lattice. Furthermore, an equivalence is established between the noncommutative semi-discrete coupled dispersionless system and a noncommutative semi-discrete sine-Gordon equation. Finally, by applying an appropriate continuum limit, we recover multisoliton solutions of the corresponding noncommutative continuous coupled dispersionless system.

## Introduction

Dispersionless integrable systems have attracted a great deal of interest due to their emergence in various areas of theoretical physics and integrable systems^1–3^. Many of these models arise as semi-classical limits of ordinary integrable systems with dispersion terms, where an appropriate scaling eliminates the dispersive contribution and produces a dispersionless hierarchy^4^. The coupled dispersionless (CD) integrable system is a prominent example with applications in theoretical physics, nonlinear waves, and engineering models such as current-fed strings in external fields^5–9^. Further developments have revealed rich solution structures, conservation laws, and geometric interpretations for CD-type systems and their generalizations^10–16^.

Noncommutative extensions of integrable systems have likewise been studied intensively in recent years^17,18^. Such models are motivated by noncommutative gauge theories on D-branes, noncommutative geometry, and by the desire to formulate integrable dynamics over non-abelian algebras^19^. A key observation is that many noncommutative integrable equations still admit exact soliton solutions, which can often be written in terms of quasideterminants^20–22^. Quasideterminants, introduced by Gelfand and Retakh as a noncommutative analogue of determinants, provide a natural algebraic language for matrix-valued solutions and have become a central tool in constructing compact representations of noncommutative soliton solutions^20,21^.

The Darboux transformation is one of the most powerful techniques for constructing exact solutions of integrable systems^23^. In the noncommutative setting, quasideterminant solutions of several integrable systems have been obtained using Darboux and binary Darboux transformations. Notable examples include the noncommutative KP equation^24,25^, the non-abelian Toda lattice and matrix sine-Gordon equation^26^, the noncommutative semi-discrete Toda equation^27^, and the noncommutative Davey–Stewartson equations^28^. Recently, quasideterminant solutions to noncommutative *q*-difference integrable equations have also been studied^29^. The matrix coupled dispersionless equations and their GBDT (generalized Bäcklund–Darboux transformation) have been investigated in^30^.

The Darboux transformation and multisoliton solutions of the commutative semi-discrete coupled dispersionless (SDCD) system were presented in^31^, where solutions were expressed in terms of ordinary determinants. The generalized coupled dispersionless system on an arbitrary non-abelian Lie group was studied by Hassan^12^, where the matrix solutions were expressed in terms of quasideterminants (see also^32–34^ and references therein). The dressing method for the generalized coupled dispersionless system was developed in^13^.

In this paper, we present a noncommutative generalization of the semi-discrete coupled dispersionless integrable system. We propose a matrix Lax pair for the noncommutative SDCD system and obtain the noncommutative equations of motion from the zero-curvature condition. We construct a Darboux transformation and express the multisoliton solutions in terms of quasideterminants. The remainder of the paper is organized as follows. “Quasideterminants” section recalls the basic properties of quasideterminants and fixes the notation used throughout the work. In “Lax representation of the noncommutative SDCD system” section, we introduce the noncommutative semi-discrete coupled dispersionless system, propose its matrix Lax pair, derive the corresponding equations of motion from the compatibility condition, and their reduction to a noncommutative semi-discrete sine-Gordon equation together with the associated continuum limit. “Darboux transformation” section is devoted to the Darboux transformation: we construct the transformation for the noncommutative semi-discrete coupled dispersionless system, iterate it to obtain multisoliton solutions in quasideterminant form, and analyse the resulting noncommutative semi-discrete matrix solutions, including their qualitative behaviour on the space–time lattice. “Concluding remarks” section contains concluding remarks and some directions for future research.

**Fig. 1:**
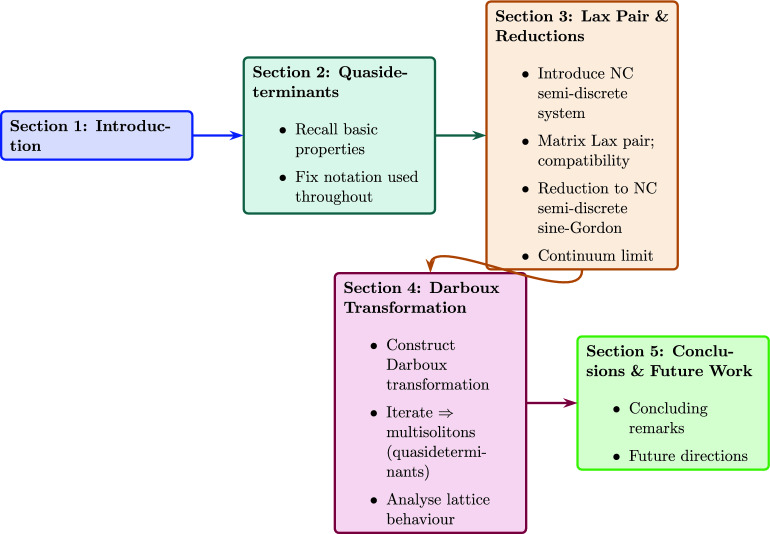
Organization of the paper.

## Quasideterminants

In this section, we recall the definition and some useful properties of quasideterminants, following^19,21,22^. Let *R* be a (not necessarily commutative) unital ring and let $A=(a_{ij})$ be an $n\times n$ matrix with entries in *R* (Fig. 1).

### Definition 2.1

Let *A* be an $n\times n$ matrix over *R* and let $1\le i,j\le n$. Denote by $A^{ij}$ the $(n-1)\times (n-1)$ submatrix obtained by deleting the *i*-th row and *j*-th column of *A*. Let $r_i^{\,j}$ denote the row vector obtained from the *i*-th row of *A* by deleting the *j*-th entry, and let $c_j^{\,i}$ denote the column vector obtained from the *j*-th column of *A* by deleting the *i*-th entry. The (*i*, *j*)-th quasideterminant of *A* is defined by

1 $$\begin{aligned} |A|_{ij}=a_{ij}-r_i^{\,j}\,(A^{ij})^{-1}c_j^{\,i}, \end{aligned}$$

provided $$(A^{ij})^{-1}$$ exists.

For a $$2\times 2$$ matrix

$$A=\begin{pmatrix} a & b\\ c & d \end{pmatrix},$$

the four quasideterminants are

2 $$\begin{aligned} |A|_{11}&= \left| \begin{array}{cc} \boxed {a} & b\\ c & d \end{array}\right| = a-bd^{-1}c, \end{aligned}$$

3 $$\begin{aligned} |A|_{12}&= \left| \begin{array}{cc} a & \boxed {b}\\ c & d \end{array}\right| = b-ac^{-1}d, \end{aligned}$$

4 $$\begin{aligned} |A|_{21}&= \left| \begin{array}{cc} a & b\\ \boxed {c} & d \end{array}\right| = c-db^{-1}a, \end{aligned}$$

5 $$\begin{aligned} |A|_{22}&= \left| \begin{array}{cc} a & b\\ c & \boxed {d} \end{array}\right| = d-ca^{-1}b. \end{aligned}$$

Here the boxed entry indicates the position (*i*, *j*) at which the quasideterminant is evaluated. In the commutative case one recovers the classical formula $|A|_{ij}=(-1)^{i+j}\det (A)/\det (A^{ij})$.

### Properties of quasideterminants

#### Proposition 2.2

(Row and column operations) *Let* *A* *be an* $n\times n$ *matrix over* *R*.

(i) *If* *B* *is obtained from* *A* *by multiplying the* *i*-*th row on the left by an invertible element* $\rho \in R$, *then* $|B|_{ij}=\rho \,|A|_{ij}$.
(ii) *If* *B* *is obtained from* *A* *by multiplying the* *j*-*th column on the right by an invertible element* $\rho \in R$, *then* $|B|_{ij}=|A|_{ij}\,\rho$.
(iii) *If* *B* *is obtained from* *A* *by adding a left multiple of the* *k*-*th row* ($k\ne i$) *to the* *i*-*th row, then* $|B|_{ij}=|A|_{ij}$ *for all* *j*.
(iv) *If* *B* *is obtained from* *A* *by adding a right multiple of the* $\ell$-*th column* ($\ell \ne j$*) to the* *j-th column, then* $|B|_{ij}=|A|_{ij}$ *for all* *i*.

These objects also satisfy several key algebraic identities which will be used later. One of the most important is the noncommutative Jacobi identity

6 $$\begin{aligned} \left| \begin{array}{ccc} R_1 & R_2 & R_3\\ R_4 & A_{11} & A_{12}\\ R_5 & A_{21} & \boxed {A_{22}} \end{array}\right| = \left| \begin{array}{cc} R_1 & R_3\\ R_5 & \boxed {A_{22}} \end{array}\right| - \left| \begin{array}{cc} R_1 & R_2\\ R_5 & \boxed {A_{21}} \end{array}\right| \left| \begin{array}{cc} R_1 & R_3\\ R_4 & \boxed {A_{11}} \end{array}\right| ^{-1} \left| \begin{array}{cc} R_1 & R_2\\ R_4 & \boxed {A_{12}} \end{array}\right| , \end{aligned}$$

where $A_{11},A_{12},A_{21},A_{22}$ are square blocks over *R* and $R_1,\dots ,R_5$ denote compatible block rows.

A closely related consequence is the homological relation

7 $$\begin{aligned} \left| \begin{array}{cc} R_1 & R_2\\ R_3 & \boxed {R_4} \end{array}\right| = \left| \begin{array}{cc} R_1 & \boxed {0}\\ R_3 & 1 \end{array}\right| ^{-1} \left| \begin{array}{cc} R_1 & \boxed {R_2}\\ R_3 & R_4 \end{array}\right| , \end{aligned}$$

which is frequently used to simplify iterated quasideterminants.

For a $$2\times 2$$ matrix

$$A= \begin{pmatrix} a_{11} & a_{12}\\ a_{21} & a_{22} \end{pmatrix},$$

the inverse can be written in terms of quasideterminants as

8 $$\begin{aligned} A^{-1}= \begin{pmatrix} \bigl (a_{11}-a_{12}a_{22}^{-1}a_{21}\bigr )^{-1} & \bigl (a_{21}-a_{22}a_{12}^{-1}a_{11}\bigr )^{-1} \\ \bigl (a_{12}-a_{11}a_{21}^{-1}a_{22}\bigr )^{-1} & \bigl (a_{22}-a_{21}a_{11}^{-1}a_{12}\bigr )^{-1} \end{pmatrix}. \end{aligned}$$

These identities provide convenient computational rules for manipulating quasideterminant expressions in the noncommutative setting.

#### Remark 2.3

In the commutative limit, where all entries commute, the quasideterminant $|A|_{ij}$ reduces to $(-1)^{i+j}\det (A)/\det (A^{ij})$, recovering the classical result. Consequently, the quasideterminant solutions presented in the subsequent sections reduce to the determinantal solutions of the commutative semi-discrete coupled dispersionless system obtained in^31^.

## Lax representation of the noncommutative SDCD system

The Lax pair of the noncommutative SDCD system is a set of differential-difference linear equations:

9 $$\begin{aligned} \Xi _{n+1}(\lambda )&= A_n(\lambda ) \, \Xi _n(\lambda ) \,, \end{aligned}$$

10 $$\begin{aligned} \frac{d}{dt} \Xi _n(\lambda )&= B_n(\lambda ) \, \Xi _n(\lambda ) \,, \end{aligned}$$

where $\Xi _n$ is a matrix-valued eigenfunction depending on the spectral parameter $\lambda$, and the matrices $A_n(\lambda )$ and $B_n(\lambda )$ are given by

11 $$\begin{aligned} A_n&= I + \lambda ^{-1}S_n = I + \dot{\imath }\lambda ^{-1} \begin{pmatrix} (\rho _{n+1} - \rho _n) & (\omega _{n+1} - \omega _n) \\ (\omega _{n+1} - \omega _n) & -(\rho _{n+1} - \rho _n) \end{pmatrix}, \end{aligned}$$

12 $$\begin{aligned} B_n&= W_n + \lambda G = \begin{pmatrix} 0 & -\omega _n \\ \omega _n & 0 \end{pmatrix} + \begin{pmatrix} -\frac{\dot{\imath }\lambda }{2} & 0 \\ 0 & \frac{\dot{\imath }\lambda }{2} \end{pmatrix}. \end{aligned}$$

Here $\rho _n, \; \omega _n$ are now matrix-valued functions in $S_n$, and *I* is the identity matrix. It is important to note that since the entries are noncommutative, the ordering of the products in the Lax pair and in the equations of motion must be carefully maintained.

The compatibility condition (zero-curvature condition) of the Lax pair (9) and (10), namely

13 $$\begin{aligned} \frac{d}{dt} A_n + A_n \, B_n - B_{n+1} \, A_n = 0 \,, \end{aligned}$$

yields the noncommutative SDCD system:

14 $$\begin{aligned} \frac{d}{dt}(\rho _{n+1} - \rho _n) + \omega _{n+1}(\omega _{n+1} - \omega _n) + (\omega _{n+1} - \omega _n) \omega _n&= 0 \,, \end{aligned}$$

15 $$\begin{aligned} \frac{d}{dt}(\omega _{n+1} - \omega _n) - \omega _{n+1}(\rho _{n+1} - \rho _n) - (\rho _{n+1} - \rho _n) \omega _n&= 0 \,. \end{aligned}$$

Note that in the commutative limit (when $\rho _n$ and $\omega _n$ are scalar-valued), the terms $\omega _{n+1}(\omega _{n+1}-\omega _n) + (\omega _{n+1}-\omega _n)\omega _n$ reduce to $(\omega _{n+1}+\omega _n)(\omega _{n+1}-\omega _n)$ and similarly for the second equation, recovering the commutative SDCD system of^31^.

### Gauge transformation

For convenience, we apply a gauge transformation to obtain an equivalent Lax representation. Let

16 $$\begin{aligned} J = \frac{1}{\sqrt{2}} \begin{pmatrix} 1 & 1 \\ -\dot{\imath } & \dot{\imath } \end{pmatrix}. \end{aligned}$$

The gauge transformation on the Lax matrices is

17 $$\begin{aligned} \widetilde{A}_n = J^{-1} A_n J \,, \qquad \widetilde{B}_n = J^{-1} B_n J \,. \end{aligned}$$

Under this transformation, the Lax pair becomes

18 $$\begin{aligned} \widetilde{\Xi }_{n+1}&= \widetilde{A}_n \, \widetilde{\Xi }_n \,, \end{aligned}$$

19 $$\begin{aligned} \frac{d}{dt} \widetilde{\Xi }_n&= \widetilde{B}_n \, \widetilde{\Xi }_n \,, \end{aligned}$$

where $\widetilde{\Xi }_n = J^{-1} \Xi _n$. If we write $$\widetilde{\Xi }_n = \begin{pmatrix} X_n \\ Y_n \end{pmatrix}^T$$, the Lax pair can be expressed in terms of the matrix-valued functions $X_n$ and $Y_n$.

### Continuum limit

In the continuum limit $b \rightarrow 0$ (where *b* is the lattice parameter), with

$$\rho _n \rightarrow \rho (x,t), \quad \rho _{n+1} \rightarrow \rho (x,t) + b\,\partial _x \rho (x,t) + O(b^2),$$

the noncommutative SDCD system reduces to the noncommutative continuous CD integrable system:

20 $$\begin{aligned} \partial _t \partial _x q + r \, \partial _x r + \partial _x r \, r&= 0 \,, \end{aligned}$$

21 $$\begin{aligned} \partial _t \partial _x r - r \, \partial _x q - \partial _x q \, r&= 0 \,, \end{aligned}$$

which is the noncommutative generalization of the continuous CD system.

### Relation with an integrable noncommutative semi-discrete sine-Gordon equation

In this section, we show that nc-SDCD system represented by Eqs. (14) and (15) is equivalent to an integrable noncommutative semi-discrete sine-Gordon (nc-SdSG) equation, subject to certain constraints.

An integrable nc-SG equation has been proposed in Ref.^35^ and is given by

22 $$\begin{aligned} \partial _t\partial _x\phi = \frac{m^2}{\beta }\,\frac{e^{i\beta \phi } - e^{-i\beta \phi }}{2i} = \frac{m^2}{\beta }\sin (\beta \phi ) \,, \end{aligned}$$

with the constraints

23 $$\begin{aligned} \partial _x e^{i\beta \phi } - \frac{i\beta }{2}\left( e^{i\beta \phi }\partial _x\phi + \partial _x\phi \, e^{i\beta \phi }\right)&= 0 \,, \end{aligned}$$

24 $$\begin{aligned} \partial _x e^{-i\beta \phi } + \frac{i\beta }{2}\left( e^{-i\beta \phi }\partial _x\phi + \partial _x\phi \, e^{-i\beta \phi }\right)&= 0 \,. \end{aligned}$$

In the continuous case^36^, the nc-CD system reduces to the nc-SG equation through the substitution $\rho _x = \frac{m^2}{2}\cos (\beta \phi )$ and $r = \frac{\beta }{2}\partial _t\phi$. We now construct the semi-discrete analogue of this reduction.

For the semi-discrete case, the nc-SdSG equation is defined as

25 $$\begin{aligned} \frac{d}{dt}(\phi _{n+1} - \phi _n) = \frac{2m^2}{\beta }\,\frac{e^{\frac{i\beta }{2}(\phi _{n+1}+\phi _n)} - e^{-\frac{i\beta }{2}(\phi _{n+1}+\phi _n)}}{2i} = \frac{2m^2}{\beta }\sin \!\left( \frac{\beta (\phi _{n+1}+\phi _n)}{2}\right) , \end{aligned}$$

where $\phi _n = \phi _n(t)$ are noncommutative functions depending on the discrete variable *n* and continuous variable *t*. We impose the following constraints on the noncommutative exponentials:

26 $$\begin{aligned} \frac{d}{dt} e^{\frac{i\beta }{2}(\phi _{n+1}+\phi _n)} - \frac{i\beta }{4}\left( e^{\frac{i\beta }{2}(\phi _{n+1}+\phi _n)}(\dot{\phi }_{n+1}+\dot{\phi }_n) + (\dot{\phi }_{n+1}+\dot{\phi }_n)\,e^{\frac{i\beta }{2}(\phi _{n+1}+\phi _n)}\right)&= 0 \,, \end{aligned}$$

27 $$\begin{aligned} \frac{d}{dt} e^{-\frac{i\beta }{2}(\phi _{n+1}+\phi _n)} + \frac{i\beta }{4}\left( e^{-\frac{i\beta }{2}(\phi _{n+1}+\phi _n)}(\dot{\phi }_{n+1}+\dot{\phi }_n) + (\dot{\phi }_{n+1}+\dot{\phi }_n)\,e^{-\frac{i\beta }{2}(\phi _{n+1}+\phi _n)}\right)&= 0 \,. \end{aligned}$$

These constraints are the semi-discrete analogues of the constraints (23) and (24), with $\partial _x$ replaced by the semi-discrete analogue and $\phi$ replaced by $\frac{1}{2}(\phi _{n+1}+\phi _n)$. They can be written as total derivatives and vanish identically in the commutative limit.

We now show that the nc-SDCD system (14)–(15) is equivalent to the nc-SdSG equation (25), subject to the constraints (26)–(27). We introduce the following substitution of the noncommutative functions $\rho _n$ and $\omega _n$:

28 $$\begin{aligned} \rho _{n+1} - \rho _n&= \frac{m^2}{2}\cos \!\left( \frac{\beta (\phi _{n+1}+\phi _n)}{2}\right) , \end{aligned}$$

29 $$\begin{aligned} \omega _n&= \frac{\beta }{2}\,\dot{\phi }_n \,, \end{aligned}$$

where $\dot{\phi }_n \equiv \frac{d}{dt}\phi _n$. By substituting (28) and (29) in the first equation of the nc-SDCD system (14), we obtain

30 $$\begin{aligned}&\frac{d}{dt}(\rho _{n+1} - \rho _n) + \omega _{n+1}(\omega _{n+1} - \omega _n) + (\omega _{n+1} - \omega _n)\omega _n \nonumber \\&\; = \frac{m^2}{4}\,\frac{d}{dt}\left( e^{\frac{i\beta }{2}(\phi _{n+1}+\phi _n)} + e^{-\frac{i\beta }{2}(\phi _{n+1}+\phi _n)}\right) + \frac{\beta ^2}{4}\left( \dot{\phi }_{n+1}(\dot{\phi }_{n+1} - \dot{\phi }_n) + (\dot{\phi }_{n+1} - \dot{\phi }_n)\dot{\phi }_n\right) . \end{aligned}$$

Now, using (25), we have

31 $$\begin{aligned} \dot{\phi }_{n+1} - \dot{\phi }_n = \frac{m^2}{\beta }\,\frac{e^{\frac{i\beta }{2}(\phi _{n+1}+\phi _n)} - e^{-\frac{i\beta }{2}(\phi _{n+1}+\phi _n)}}{i} \,. \end{aligned}$$

Substituting (31) into (30):

32 $$\begin{aligned}&\frac{\beta ^2}{4}\left( \dot{\phi }_{n+1}(\dot{\phi }_{n+1} - \dot{\phi }_n) + (\dot{\phi }_{n+1} - \dot{\phi }_n)\dot{\phi }_n\right) \nonumber \\&= \frac{m^2}{4}\bigg \{\frac{d}{dt}e^{\frac{i\beta }{2}(\phi _{n+1}+\phi _n)} - \frac{i\beta }{4}\Big (e^{\frac{i\beta }{2}(\phi _{n+1}+\phi _n)}(\dot{\phi }_{n+1}+\dot{\phi }_n) + (\dot{\phi }_{n+1}+\dot{\phi }_n)\,e^{\frac{i\beta }{2}(\phi _{n+1}+\phi _n)}\Big ) \nonumber \\&\quad - \frac{i\beta }{4}\Big (\dot{\phi }_{n+1}\,e^{\frac{i\beta }{2}(\phi _{n+1}+\phi _n)} - e^{\frac{i\beta }{2}(\phi _{n+1}+\phi _n)}\dot{\phi }_{n+1} + \dot{\phi }_n\,e^{\frac{i\beta }{2}(\phi _{n+1}+\phi _n)} - e^{\frac{i\beta }{2}(\phi _{n+1}+\phi _n)}\dot{\phi }_n\Big ) \nonumber \\&\quad + \frac{d}{dt}e^{-\frac{i\beta }{2}(\phi _{n+1}+\phi _n)} + \frac{i\beta }{4}\Big (e^{-\frac{i\beta }{2}(\phi _{n+1}+\phi _n)}(\dot{\phi }_{n+1}+\dot{\phi }_n) + (\dot{\phi }_{n+1}+\dot{\phi }_n)\,e^{-\frac{i\beta }{2}(\phi _{n+1}+\phi _n)}\Big ) \nonumber \\&\quad + \frac{i\beta }{4}\Big (\dot{\phi }_{n+1}\,e^{-\frac{i\beta }{2}(\phi _{n+1}+\phi _n)} - e^{-\frac{i\beta }{2}(\phi _{n+1}+\phi _n)}\dot{\phi }_{n+1} + \dot{\phi }_n\,e^{-\frac{i\beta }{2}(\phi _{n+1}+\phi _n)} - e^{-\frac{i\beta }{2}(\phi _{n+1}+\phi _n)}\dot{\phi }_n\Big )\bigg \} . \end{aligned}$$

The first two lines are precisely the constraint (26), and the last two lines correspond to the constraint (27). Therefore, when the constraints (26) and (27) are satisfied, the expression (32) vanishes, and the first equation of motion (14) is identically satisfied. Similarly, using the same constraints, it can be shown that the second equation of motion (15) is also identically satisfied.

#### Remark 3.1

In the commutative limit, the constraints (26)–(27) are automatically satisfied since $\frac{d}{dt}e^{i\beta \Xi } = i\beta \,e^{i\beta \Xi }\,\dot{\Xi }$ when all variables commute. The substitution (28)–(29) reduces to

$$\rho _{n+1} - \rho _n = \cos \!\left( \frac{\phi _{n+1}+\phi _n}{2}\right) , \qquad \omega _n = -\frac{1}{2}\dot{\phi }_n \,,$$

(with $m^2 = 2$ and $\beta = -1$), which is exactly the commutative substitution used in Ref.^31^ (Eq. (2.29)).

#### Remark 3.2

In the continuum limit $b \rightarrow 0$, with $\phi _n \rightarrow \phi (x,t)$ and $\phi _{n+1} \rightarrow \phi (x,t) + b\,\partial _x\phi (x,t) + O(b^2)$:

- The nc-SdSG equation (25) reduces to the nc-SG equation (22).
- The substitution (28) reduces to $\rho _x = \frac{m^2}{2}\cos (\beta \phi )$ and the constraints (26)–(27) reduce to the constraints (23)–(24).
- The entire reduction recovers the continuous nc-CD $\rightarrow$ nc-SG equivalence of Ref.^36^.

## Darboux transformation

Darboux transformation is a powerful solution-generating technique widely used in soliton theory^23^. In the noncommutative setting, the Darboux transformation naturally leads to solutions expressed in terms of quasideterminants rather than ordinary determinants^19,24,26^.

In this section, we construct the Darboux transformation for the noncommutative SDCD system and express the iterated solutions in terms of quasideterminants.

Let $\widetilde{\Xi }_n$ be a solution of the Lax pair (18)–(19). We define the Darboux matrix $D_n(t;\lambda )$ such that the Darboux transformation on the eigenfunction is given by

33 $$\begin{aligned} \widetilde{\Xi }_n[1] \equiv D_n(t;\lambda ) \, \widetilde{\Xi }_n = (\lambda I - \Sigma _n(t)) \, \widetilde{\Xi }_n \,, \end{aligned}$$

where *I* is the identity matrix and $\Sigma _n(t)$ is a matrix to be determined. For our system, $\Sigma _n$ is defined as

34 $$\begin{aligned} \Sigma _n = U_n \, \Lambda \, U_n^{-1} \,, \end{aligned}$$

where $\Lambda = \textrm{diag}(\lambda _1, \lambda _2)$ and $U_n$ is an invertible $2 \times 2$ matrix constructed from particular column solutions to the Lax pair at the spectral parameters $\lambda _1$ and $-\lambda _1$:

35 $$\begin{aligned} U_n = \left( |m_1\rangle _n, \, |m_2\rangle _n \right) = \begin{pmatrix} X_{n,1} & X_{n,1} \\ Y_{n,1} & -Y_{n,1} \end{pmatrix}. \end{aligned}$$

Based on this ansatz, we make the following theorem.

### Theorem 4.1

*Suppose* $(U_{n,1}, \; U_{n,2},\dots ,U_{n,k})$ *denotes specific* $m\times m$ *matrix solutions of the semi-discrete Lax pair*

36 $$\begin{aligned} \Xi _{n+1} = (I_m+\lambda ^{-1} S_n)\Xi _n,\qquad \frac{d}{dt}\Xi _n = (\lambda G + W_n)\Xi _n, \end{aligned}$$

*corresponding to eigenvalue matrices* $(\Lambda _1,\Lambda _2,\dots ,\Lambda _k)$, *where*

$$\Lambda _j=\textrm{diag}(\lambda _{j,1},\dots ,\lambda _{j,m}), \qquad j=1,\dots ,k.$$

*The* *k*–*fold application of the Darboux transformation, written in inverse–matrix form, can be expressed as*

37 $$\begin{aligned} S_n^{[k]} = S_n + (\Delta _n-I_m) \left| \begin{array}{cc} F_n & E^{(k)}\\ F_n^{(k)} & \boxed {O_m} \end{array} \right| , \end{aligned}$$

*where* $\Delta _n$ *is the shift operator* $\Delta _n f_n = f_{n+1}$, *and* $O_m$ *is the* $m\times m$ *zero matrix, the block column*

$$E^{(k)}=\begin{pmatrix} O_m&O_m&\cdots&O_m&I_m\end{pmatrix}^{\!T}\in \mathbb {C}^{mk\times m},$$

*and*

$$F_n^{(s)}=\bigl (U_{n,1}\Lambda _1^{s}, \; U_{n,2}\Lambda _2^{s}, \; \dots , \; U_{n,k}\Lambda _k^{s}\bigr ), \qquad s=0,1,\dots ,k,$$

*while the* $mk\times mk$ *block matrix* $F_n$ *is given by*

38 $$\begin{aligned} F_n= \begin{pmatrix} F_n^{(0)}\\ F_n^{(1)}\\ \vdots \\ F_n^{(k-1)} \end{pmatrix} = \begin{pmatrix} U_{n,1} & U_{n,2} & \cdots & U_{n,k}\\ U_{n,1}\Lambda _1 & U_{n,2}\Lambda _2 & \cdots & U_{n,k}\Lambda _k\\ \vdots & \vdots & \ddots & \vdots \\ U_{n,1}\Lambda _1^{(k-1)} & U_{n,2}\Lambda _2^{(k-1)} & \cdots & U_{n,k}\Lambda _k^{(k-1)} \end{pmatrix}. \end{aligned}$$

*Here* $|\;\cdot \;|$ *denotes the quasideterminant taken with respect to the lower–right* $m\times m$ *block. The transformed matrix* $S_n^{[k]}$ *has the same* $m\times m$ *structure as* $S_n$, *and the associated Lax pair* (36) *is covariant under this* *k*–*fold Darboux transformation*.

To express multi-soliton solutions for the nc-SDCD system, we arrange the eigenfunctions of the semi-discrete Lax pair in an $mk\times mk$ quasi-Wronskian matrix. For the *i*-th eigenvalue $\lambda _i$ we set

$$U_{n,\; i} = \begin{pmatrix} X_{n,\; m i-1} & X_{n,\;m i}\\ Y_{n, \; m i-1} & Y_{n, \; m i} \end{pmatrix},\qquad i=1,\dots ,k,$$

where $X_{n, m i-1},\; Y_{n,m i-1}$ solve the Lax equations at $\lambda =\lambda _{m-i}$ and $X_{m i,\; n},\; Y_{m i,\; n}$ correspond to $\lambda =\lambda _m$ (noncommutative case). Suppose

$$\widehat{V}_n = \bigl (U_1,\,U_2,\,\dots ,\,U_k\bigr )$$

be the $mk\times mk$ matrix of eigenfunctions and let $c_{mk-1},c_{mk}\in \mathbb {C}^{mk}$ be unit column vectors with 1 in positions $mk-1$ and *mk*, respectively.

The multi-soliton fields $\rho _n^{[k+1]}$ and $\omega _n^{[k+1]}$ of the noncommutative semi-discrete coupled dispersionless system are then given by

39 $$\begin{aligned} \rho _n^{[k+1]} = \rho _n - i\! \left| \begin{array}{cc} \widehat{V}_n & c_{mk-1}\\ X_n^{(k)} & \boxed {0} \end{array} \right| , \qquad \omega _n^{[k+1]} = \omega _n - i \left| \begin{array}{cc} \widehat{V}_n & c_{mk}\\ X_n^{(k)} & \boxed {0} \end{array} \right| . \end{aligned}$$

In the commutative limit, the quasideterminants in (39) reduce to ratios of determinants and reproduce the classical Wronskian-type semi-discrete soliton solutions.

### Noncommutative semi-discrete solutions

In the noncommutative setting, we keep the $2\times 2$ Lax pair of the nc-SDCD system and allow all field variables and eigenfunctions to take values in a noncommutative matrix algebra. For the one-soliton case ($k=1$), the dressed fields $\rho _n^{[2]}$ and $\omega _n^{[2]}$ (denoted by $\rho _{n}^{1}$ and $\omega _{n}^{1}$) are written in terms of $2\times 2$ quasideterminants, in complete analogy with the continuous NC case of^37^.

We choose a simple matrix seed solution

$$\omega _n = O_{2},\qquad \rho _n = c\,I_{2},$$

where *c* is a constant and $O_{2}$, $I_{2}$ denote the $2\times 2$ zero and identity matrices, respectively. The corresponding eigenfunctions of the semi-discrete Lax pair are denoted by $X_{n,\; p}(t)$ and $Y_{n, \; p}(t)$ ($p=1, \; 2$); they are obtained from the linear problem (9)-(10). Their explicit expressions will be used later for plots but are not needed in the structural formulas below.

**Quasideterminant representation.** Following the quasi-Wronskian construction, the dressed fields take the form

40 $$\begin{aligned} \omega _{n}^{1} = \omega _n - i\,D_{r,n}, \qquad \rho _{n}^{1} = \rho _n - i \,D_{q,n}, \end{aligned}$$

The matrices $D_{r,n}$ and $D_{q,n}$ are $2\times 2$ quasideterminants defined by41

Here $\widehat{V}_n$ is a $4\times 4$ matrix built from the eigenfunctions at the chosen spectral parameter, and $c_{pq}$ ($p,q=1,2$) are canonical basis vectors in $\mathbb {C}^{4}$,

$$c_{11}=(1,\; 0, \; 0, \; 0)^{T},\quad c_{12}=(0, \; 1, \; 0, \; 0)^{T},\quad c_{21}=(0, \;0, \;1, \; 0)^{T},\quad c_{22}=(0, \; 0, \; 0, \; 1)^{T},$$

while $C_{11}$ and $C_{12}$ are $1\times 4$ rows containing the constant coefficients and eigenfunctions (semi-discrete analogues of $C_{11},C_{12}$ in^37^).

The matrix $\widehat{V}_n$ is given by

$$\widehat{V}_n = \begin{pmatrix} \kappa _{11} X_{n ,\; 1} & \kappa _{12} & -\gamma _{11} X_{n,\;2} & -\gamma _{12}\\ \kappa _{21} & \kappa _{22} X_{n,\;1} & -\gamma _{21} & -\gamma _{22} X_{n,\;2}\\ \beta _{11} Y_{n,\;1} & \beta _{12} & \xi _{11} Y_{n,\;2} & \xi _{12}\\ \beta _{21} & \beta _{22} Y_{n,\;1} & \xi _{21} & \xi _{22} Y_{n,\;2} \end{pmatrix},$$

where $\kappa _{ij}, \; \gamma _{ij}, \; \beta _{ij}, \; \xi _{ij}$ are constants. Writing

$$\omega _{n}^{1} \equiv \begin{pmatrix} \omega _{n}^{11} & \omega _{n}^{12}\\ \omega _{n}^{21} & \omega _{n}^{22} \end{pmatrix}, \qquad \rho _{n}^{1} \equiv \begin{pmatrix} \rho _{n}^{11} & \rho _{n}^{12} \\ \rho _{n}^{21} & \rho _{n}^{22} \end{pmatrix},$$

each entry is obtained from (41) by taking the corresponding $2\times 2$ quasideterminant. For instance,

$$\omega _{n}^{11} = -i \left| \begin{array}{cc} \widehat{V}_n & c_{21}\\ C_{11} & 0 \end{array}\right| , \qquad \rho _{n}^{11} = c - i \left| \begin{array}{cc} \widehat{V}_n & c_{11}\\ C_{11} & 0 \end{array}\right| ,$$

and similar expressions hold for $\omega _{n}^{12}, \; \omega _{n}^{21}, \; \omega _{n}^{22}$ and $\rho _{n}^{12}, \; q^{21}_{n},q^{22}_{n}$. Here the diagonal entries $\omega ^{11}_n$, $\omega ^{22}_n$ and $\rho ^{11}_n$, $\rho ^{22}_n$ represent the dominant (self-interaction) mode of each polarization channel, while the off-diagonal entries $\omega ^{12}_n$, $\omega ^{21}_n$ and $\rho ^{12}_n$, $\rho ^{21}_n$ encode the cross-coupling or mode-conversion amplitude between the two internal degrees of freedom introduced by noncommutativity. In the commutative limit the off-diagonal components vanish and the two fields decouple, reproducing the scalar semi-discrete soliton of Ref.^31^. These formulas provide compact quasideterminant representations of noncommutative semi-discrete one-soliton solutions. In the commutative limit, the quasideterminants reduce to ratios of ordinary determinants and reproduce the semi-discrete scalar solitons obtained earlier.

**Fig. 2:**
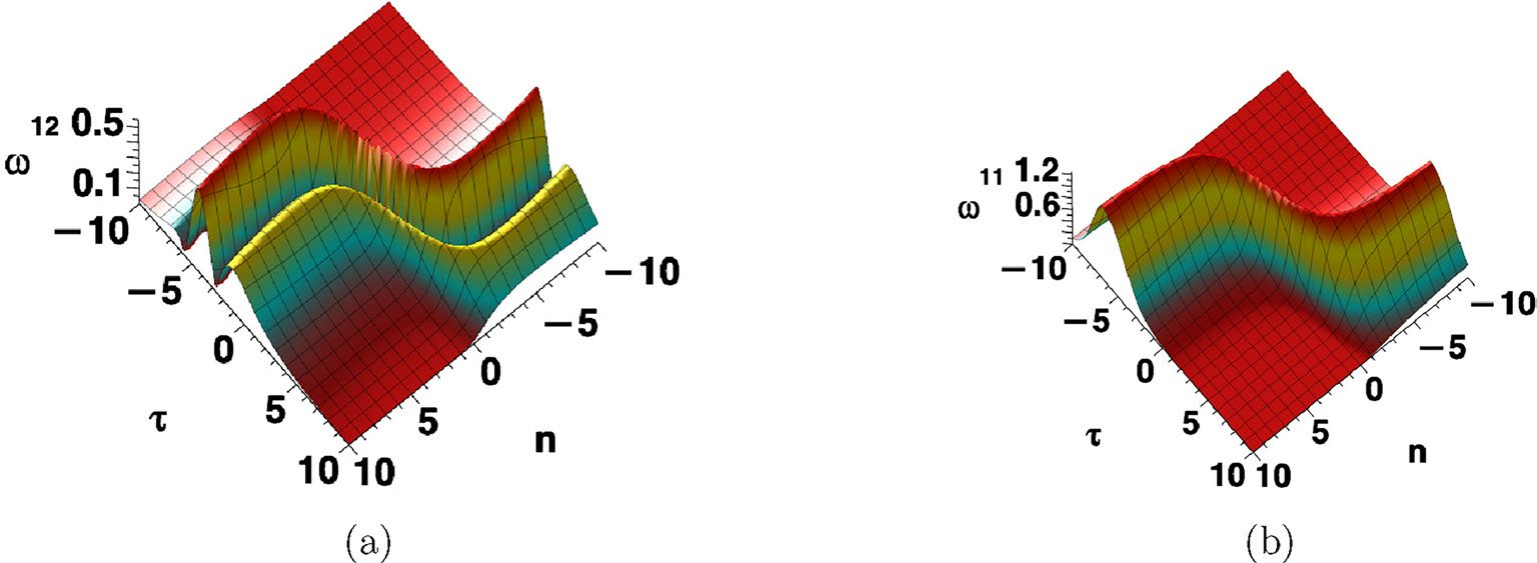
Three–dimensional profiles of the matrix field $\omega _n^{[1]}$ for the parameter set $a=-0.15$, $c=0.2$, $\varepsilon =0.1$, $\lambda _1=-1.9i$, $\lambda _2=1.9i$, $B_1=0.2$, $B_2=-0.2$, $x=0$ and $\kappa _{11}=1$, $\kappa _{12}=0.5$, $\kappa _{21}=0$, $\kappa _{22}=1$, $\beta _{11}=1$, $\beta _{12}=2$, $\beta _{21}=-2.2$, $\beta _{22}=1$, $\gamma _{11}=0.9$, $\gamma _{12}=0.1$, $\gamma _{21}=0$, $\gamma _{22}=1$, $\zeta _{11}=1$, $\zeta _{12}=0$, $\zeta _{21}=0$, $\zeta _{22}=1$. Here the horizontal axes are the discrete lattice index *n* (denoted by *y* in the plots) and the rescaled time variable $\tau$.

To visualise the qualitative behaviour of the noncommutative semi-discrete one–soliton solution constructed above, we now plot representative components of the dressed fields $\omega _n^{[1]}$ and $\rho _n^{[1]}$. In all plots the horizontal axes correspond to the discrete lattice index *n* (denoted by *y* in the graphics) and to the rescaled time variable $\tau$, while the vertical axis shows the amplitude of a particular matrix entry. The parameter set is fixed to

$$c=-0.15,\quad \quad \varepsilon =0.1,\quad \lambda _1=-1.9i,\ \lambda _2=1.9i,$$

together with the constants

$$(\kappa _{ij}),\ (\beta _{ij}),\ (\gamma _{ij}),\ (\zeta _{ij})$$

given in the caption of Fig. 2. This choice produces a single localized pulse on the lattice and allows us to distinguish clearly between diagonal and off–diagonal matrix components in the noncommutative regime.

**Fig. 3:**
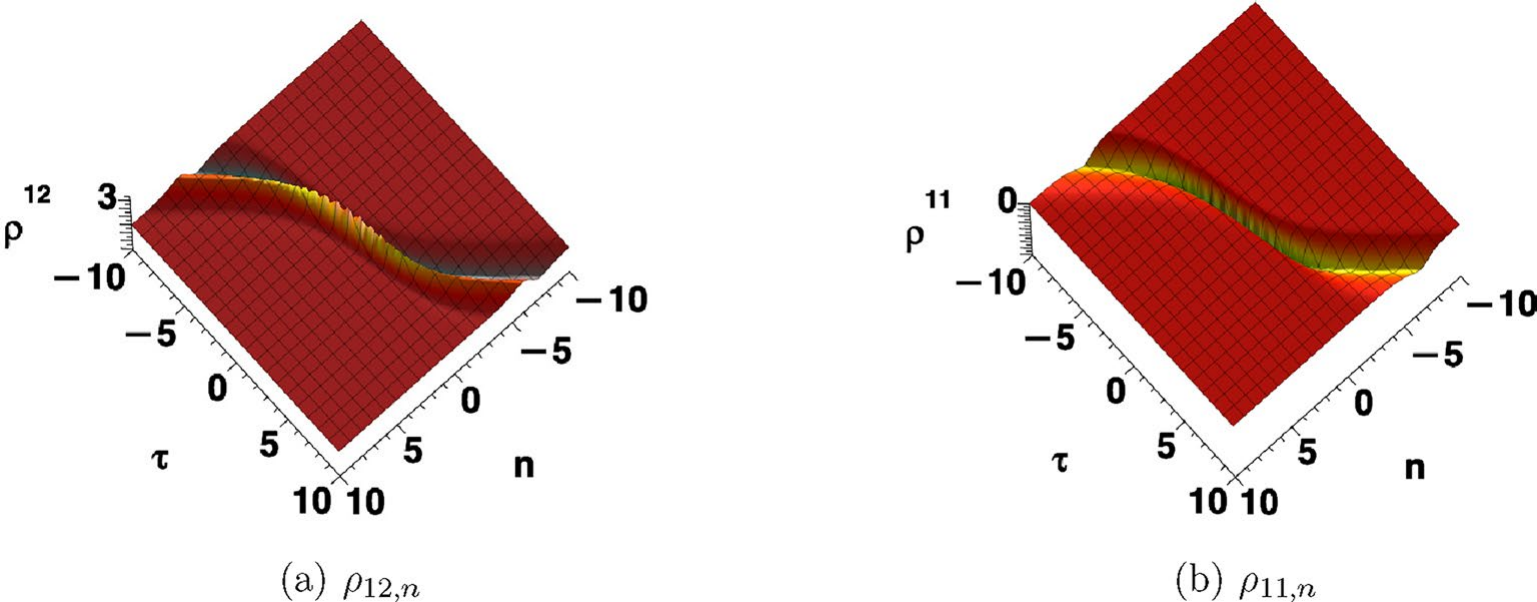
Profiles of the potential-type field $\rho _n^{[1]}$ for the parameter set $a=-0.15$, $c=0.2$, $\varepsilon =0.1$, $\lambda _1=-1.9i$, $\lambda _2=1.9i$, $B_1=0.2$, $B_2=-0.2$, $x=0$ and $\kappa _{11}=1$, $\kappa _{12}=0.5$, $\kappa _{21}=0$, $\kappa _{22}=1$, $\beta _{11}=1$, $\beta _{12}=2$, $\beta _{21}=-2.2$, $\beta _{22}=1$, $\gamma _{11}=0.9$, $\gamma _{12}=0.1$, $\gamma _{21}=0$, $\gamma _{22}=1$, $\zeta _{11}=1$, $\zeta _{12}=0$, $\zeta _{21}=0$, $\zeta _{22}=1$. Here the horizontal axes are the discrete lattice index *n* (denoted by *y* in the plots) and the rescaled time variable $\tau$.

The numerical computations and surface plots in Figs. 2, 3 were generated using Maple. These surfaces illustrate how the noncommutative semi-discrete one–soliton solution is distributed between diagonal and off–diagonal components of the $2\times 2$ matrix fields. In Fig. 2a, the off–diagonal entry $\omega _{n}^{12}$ forms a narrow bright strip which propagates in the $(n, \; \tau )$–plane along a curved trajectory. The amplitude remains relatively small compared with the diagonal part, indicating that noncommutative coupling generates a localized but weak energy transfer between the two internal modes. By contrast, Fig. 2b shows that $\omega ^{11}_{n}$ supports a broad soliton–like ridge with significantly larger peak value and smooth tails in both directions of *n*. This behaviour confirms that the main portion of the excitation remains trapped in a single polarization channel, while the off–diagonal entry primarily encodes mode–conversion effects.

The potential-type field $\rho _n^{1}$ exhibits a complementary structure. In Fig. 3a, the component $\rho ^{12}_{n}$ develops a sharp tilted interface where the amplitude changes rapidly in the neighbourhood of the soliton centre, whereas away from this interface the field is almost constant. This configuration can be interpreted as a localized phase or refractive–index jump induced by the Darboux transformation in the noncommutative setting. Figure 3b reveals that $\rho ^{11}_{n}$ combines a wide dark depression, measured relative to the constant background $c = -0.15$, with a narrow oscillatory ridge aligned with the centre of the soliton. The coexistence of dark and bright features in the same matrix entry is a typical signature of noncommutative dressing, and it disappears in the commutative limit where the quasideterminants reduce to ordinary determinant ratios.

From a physical point of view, the present semi-discrete model may describe wave propagation in a birefringent optical lattice, a coupled spin chain or a magneto–elastic array, where the site index *n* labels the waveguide (or spin) and $\tau$ plays the role of an effective time variable. In this interpretation, the diagonal components $\omega ^{11}_{n}$ and $\rho ^{11}_{n}$ represent the dominant polarization, supporting a robust travelling pulse whose centre follows a slightly bent trajectory due to the lattice inhomogeneity encoded by *a* and by the coefficient structure of the Lax pair. The off–diagonal components $\omega ^{12}_{12}$ and $\rho ^{12}_{n}$ then measure the strength of cross–coupling between the two polarizations. Their localized ridges indicate that energy is periodically exchanged between neighbouring channels during the soliton evolution, while the overall profile remains stable. Such behaviour is relevant for all–optical switching and controlled mode conversion in nonlinear waveguide arrays, as well as for magnonic or spintronic devices where information is carried by localized excitations on discrete lattices.

## Concluding remarks

In this work we have developed a noncommutative generalization of the semi-discrete coupled dispersionless system and analysed it through a matrix Lax pair and Darboux transformation. The compatibility condition of the semi-discrete Lax pair yields a noncommutative system of equations of motion, which admits a natural formulation in terms of matrix-valued fields. By constructing an explicit Darboux transformation and iterating it, we obtained compact quasideterminant representations of multisoliton solutions. These quasideterminant formulae remain valid in the fully noncommutative setting and reduce to determinant expressions in the commutative limit, thereby recovering known semi-discrete coupled dispersionless solitons.

A further outcome of the analysis is the identification of a noncommutative semi-discrete sine–Gordon equation that is equivalent to the noncommutative semi-discrete coupled dispersionless system under an appropriate reduction. This equivalence is significant as it establishes a direct bridge between two important classes of noncommutative integrable systems, extending the known continuous correspondence to the semi-discrete setting. The semi-discrete solutions of the matrix fields were examined in detail, the diagonal entries $\omega ^{11}_n$, $\omega ^{22}_n$ and $\rho ^{11}_n$, $\rho ^{22}_n$ capture the dominant polarization dynamics, while the off-diagonal entries $\omega ^{12}_n$, $\omega ^{21}_n$ and $\rho ^{12}_n$, $\rho ^{21}_n$ encode the mode-conversion effects induced by noncommutativity. Finally, by taking a suitable continuum limit, we showed how the same framework produces multisoliton solutions of the corresponding noncommutative continuous coupled dispersionless system.

The present study suggests several avenues for further investigation:

- Extend the construction to multi-component or higher-rank noncommutative semi-discrete coupled dispersionless systems and examine the rich family of vector and matrix solitons that may arise.
- Analyse conservation laws, Hamiltonian structures and symmetry reductions for the noncommutative semi-discrete coupled dispersionless system and for its sine–Gordon-type reductions.
- Investigate numerical schemes that preserve the underlying integrable structure and compare their performance with the exact quasideterminant solutions on long time scales.
- Explore physical applications of the noncommutative semi-discrete models to birefringent optical lattices, spin-chain arrays or other discrete media where matrix-valued wave amplitudes and weak noncommutativity may play a role.
- Study higher-order or fractional generalizations, as well as possible extensions to $(2+1)$-dimensional noncommutative semi-discrete dispersionless systems.

## Data Availability

All data generated or analysed during this study are included in this article.
